# Analysis of the protein-protein interaction networks of differentially expressed genes in pulmonary embolism

**DOI:** 10.3892/mmr.2014.3006

**Published:** 2014-11-26

**Authors:** HAO WANG, CHEN WANG, LEI ZHANG, YINGHUA LU, QIANGLIN DUAN, ZHU GONG, AIBIN LIANG, HAOMING SONG, LEMIN WANG

**Affiliations:** 1 Department of Family Medicine, Tongji Hospital, School of Medicine, Tongji University, Shanghai 200065, P.R. China; 2 Department of Cardiology, Tongji Hospital, School of Medicine, Tongji University, Shanghai 200065, P.R. China; 3 Department of Hematology, Tongji Hospital, School of Medicine, Tongji University, Shanghai 200065, P.R. China

**Keywords:** pulmonary embolism, protein-protein interaction network, functional modules, differentially expressed genes, pathways

## Abstract

The aim of the present study was to explore the function and interaction of differentially expressed genes (DEGs) in pulmonary embolism (PE). The gene expression profile GSE13535, was downloaded from the Gene Expression Omnibus database. The DEGs 2 and 18 h post-PE initiation were identified using the affy package in R software. The Kyoto Encyclopedia of Genes and Genomes (KEGG) pathways of the DEGs were analyzed using Database for Annotation Visualization and Integrated Discovery (DAVID) online analytical tools. In addition, protein-protein interaction (PPI) networks of the DEGs were constructed using the Search Tool for the Retrieval of Interacting Genes/Proteins. The PPI network at 18 h was modularized using ClusterONE, and a functional enrichment analysis of the DEGs in the top three modules was performed with DAVID. Overall, 80 and 346 DEGs were identified 2 and 18 h after PE initiation, respectively. The KEGG pathways, including chemokine signaling and toll-like receptor signaling, were shown to be significantly enriched. The five highest degree nodes in the PPI networks at 2 or 18 h were screened. The module analysis of the PPI network at 18 h revealed 11 hub nodes. A Gene Ontology terms analysis demonstrated that the DEGs in the top three modules were associated with the inflammatory, defense and immune responses. The results of the present study suggest that the DEGs identified, including chemokine-related genes TFPI2 and TNF, may be potential target genes for the treatment of PE. The chemokine signaling pathway, inflammatory response and immune response were explored, and it may be suggested that these pathways have important roles in PE.

## Introduction

After cancer and myocardial infarction, pulmonary embolism (PE) is the third-most-common cause of mortality in the United States (1,2), with >600,000 cases arising annually (3). The one-year mortality rate is 2.5% for treated PE (4) and 30% for untreated PE (5), according to previous research. Numerous studies have focused on the causes of PE. PE has been identified as the most serious complication of deep vein thrombosis (DVT) (6), and is defined as an embolus obstructing a vessel, or the outline of an embolus within a vessel (7,8). DVT is closely associated with PE (9), as well as cardiac disease (10) and other chronic diseases (4). The presenting symptoms of PE are non-specific and final diagnosis is based on an angiogram (4).

Previous studies have identified genetic mutations that are associated with PE, such as Factor V Leiden (11), prothrombin factor II G20210A (12) and val34leu mutation in factor XIII (13). In addition, common polymorphisms of methylenetetrahydrofolate reductase (14,15) appear to be associated with PE. Genes, such as plasminogen activator inhibitor-1 (PAI-1) that reduces fibrinolytic capacity (16) and granulocyte-macrophage colony stimulating factor (GM-CSF) that participates in the immune function by affecting the balance of Th (helper T cell) 1/Th2 (14,17), are reported as risk factors for PE. Furthermore, deficiencies of certain proteins, such as hereditary protein S (16), protein C and antithrombin III (12), have been reported to impact arterial thrombosis and PE. Although previous studies have identified numerous potential genes and proteins that may be the determinants of PE, it remains essential to research the potential etiology and pathogenesis of PE, due to its high incidence, high rate of mortality, high level of misdiagnosis and low detection rate.

The present study used microarrays to identify differentially expressed genes (DEGs) between specimens acquired 2 and 18 h following injection of a microsphere, and specimens acquired following injection of a control vehicle. Bioinformatics methods were used to construct protein-protein interaction (PPI) networks, and the functional modules in the networks were analyzed.

## Materials and methods

### Derivation of genetic data

The gene expression profile of GSE13535 (18) was downloaded from the Gene Expression Omnibus (GEO) database (http://www.ncbi.nlm.nih.gov/geo/query/acc.cgi?acc=GSE13535), a public functional genomics data repository. The GSE13535 expression profile is based on the GPL1355 platform ([Rat230_2] Affymetrix Rat Genome 230 2.0 Array). The specimens were acquired at 2 and 18 h after microsphere-injection in a rat model of PE (18), and the vehicle-treated samples were considered a control group. A total of 22 specimens were available for the present study, including three vehicle-treated specimens acquired at 2 h, eight microsphere-injection specimens acquired at 2 h, three vehicle-treated specimens acquired at 18 h and eight microsphere-injection specimens acquired at 18 h.

### Data preprocessing and screening of DEGs

The derived genetic data was initially analyzed using affy package (19) in R software, and the array files were converted into gene expression spectrum data. Robust multi-array average (20) was then used to standardize the expression spectrum data. Furthermore, the limma package (21) was used to calculate and analyze the DEGs of the sample groups, as compared with the control group, and the Bayes method was applied for a multiple range test. The DEGs were screened based on a cut-off value, which was set at |logFC| (fold change)>1 and P<0.05.

### Kyoto Encyclopedia of Genes and Genomes (KEGG) pathways enrichment analysis

Database for Annotation Visualization and Integrated Discovery (DAVID) (22) online analytical tools were used to perform KEGG pathway analysis on the DEGs obtained from the experimental specimens. The pathways with a P<0.05 were considered to indicate significant pathways in which the DEGs were involved.

### PPI network construction

The PPI networks of the DEGs at 2 and 18 h after PE initiation were constructed from PPI pairs whose protein interaction scores were >0.4, as determined by the Search Tool for the Retrieval of Interacting Genes/Proteins (23) online tool. The protein interaction scores were calculated using the following formula (24):

$$\begin{array}{l} S\left(\text{e}\left(x,y\right)\right)=f\left(diff\left(x\right),corr\left(x,y\right),diff\left(y\right)\right)\\ =-2\sum_{i=1}^{k}{\text{log}}_{e}\left(p_{i}\right) \end{array}$$

Where diff (*x*) and diff (*y*) are differential expression assessments of gene x and gene y, respectively. Corr (*x*, *y*) represents the correlation between gene *x* and gene *y*. k=3, p_1_ and p_2_ are the P-values of the differential expression of the two nodes, and p_3_ is the P-value of their co-expression (24). Visualization of the PPI network was acquired using the igraph package (25) in R software.

### Functional module analysis

Functional modules of the networks were explored using the ClusterONE plug-in of Cytoscape software (26). The top three modules of the network at 18 h post-PE initiation were screened under the condition of minimum size=6 and minimum density=0.05. The DEGs in the top three modules were then analyzed by Gene Ontology (GO) functional enrichment analysis.

## Results

### Screening DEGs at two time points

**Figure 1:**
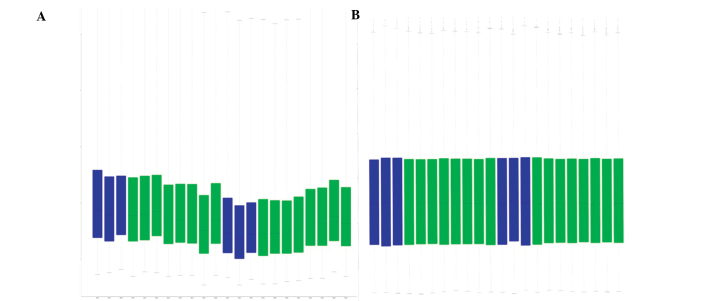
Box plots of the expression spectrum data (A) before and (B) after standardization. Blue represents the control group and green represents the experimental groups.

**Figure 2:**
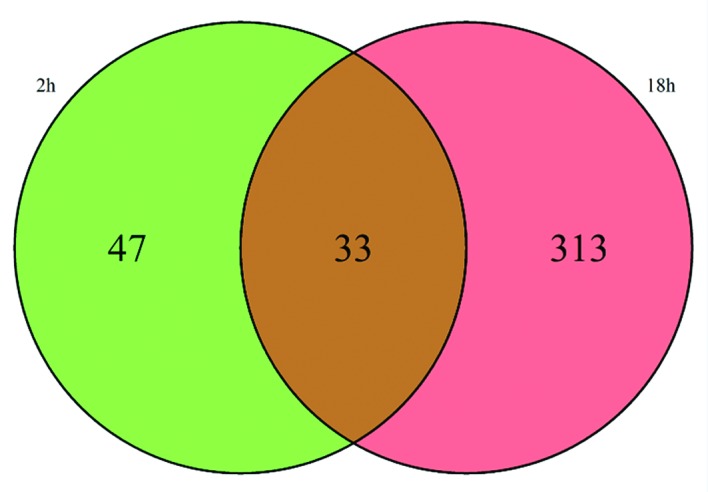
Venn diagram of the differentially expressed genes (DEGs) identified 2 and 18 h after pulmonary embolism (PE) initiation. Green represents the DEGs of PE at 2 h, red represents the DEGs of PE at 18 h, and brown represents the DEGs observed at both 2 and 18 h.

The standardization of the expression spectrum data showed a good result (Fig. 1). A total of 80 and 346 DEGs were identified 2 and 18 h following injection with the microsphere, respectively, as compared with the vehicle-treated specimens. There were 47 DEGs specifically at 2 h, 313 DEGs specifically at 18 h, and 33 genes that were differentially expressed at 2 and 18 h (Fig. 2). The most significantly upregulated and downregulated genes at 2 h were chemokine (C-C motif) ligand (CCL) 2 and Retnla (resistin like α), and at 18 h were tissue factor pathway inhibitor 2 (TFPI2) and cytochrome P450 family 2 subfamily E polypeptide 1 (Cyp1a1).

### Pathway analysis of DEGs

**Figure 3:**
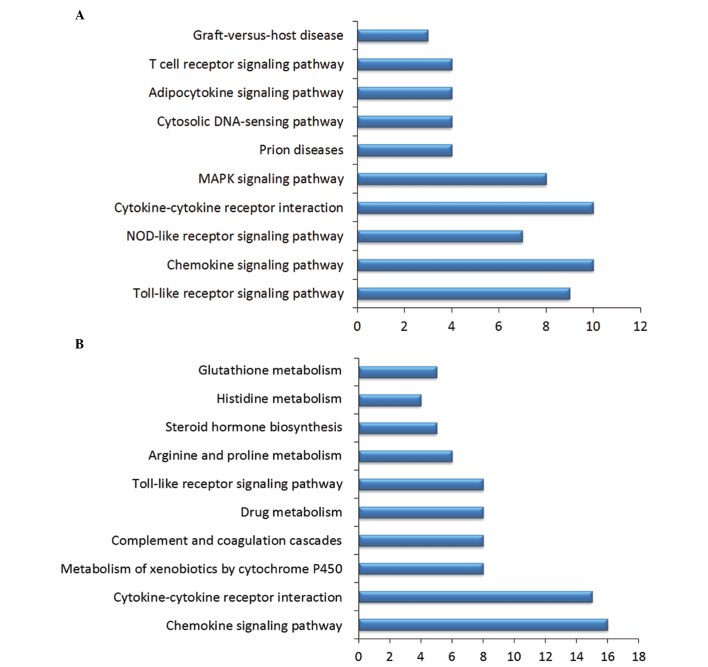
The enriched Kyoto Encyclopedia of Genes and Genomes (KEGG) pathways of the differentially expressed genes (DEGs) in pulmonary emblolism (PE) specimens at the different time points: (A) 2 h and (B) 18 h. The X-axis represents the amounts of DEGs, and the Y-axis represents enriched KEGG pathways. The blue bars represent the P-value, which was determined using the Database for Annotation Visualization and Integrated Discovery. The P value is increased from the bottom up.

The KEGG pathway analysis at 2 h after PE initiation, identified DEGs that were significantly enriched in the toll-like receptor signaling pathway, chemokine signaling pathway, cytokine-cytokine receptor interaction and Ras-mitogen-activated protein kinase (MAPK) signaling pathway (Fig. 3A). Two of these pathways, the chemokine signaling pathway and the cytokine-cytokine receptor interaction pathway, were also enriched in the 18 h group (Fig. 3B).

### Results of the PPI network

**Figure 4:**
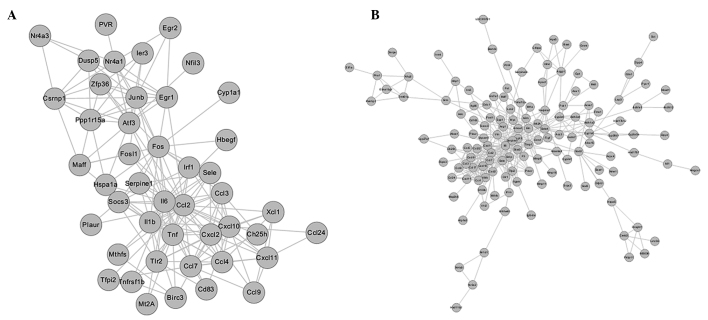
Protein-protein interaction networks of the differentially expressed genes (DEGs) in pulmonary embolism at the different time points (A) 2 h and (B) 18 h. The nodes indicate the DEGs and the edges indicate the interactions between two genes.

The PPI network of DEGs 2 h after PE initiation consisted of 45 nodes and 170 edges, whereas 137 nodes and 378 edges were included in the PPI network of the 18 h group (Fig. 4). In the network of DEGs from the 2 h group, the top five highest degree nodes (Fig. 4A) included CCL2, interleukin (IL)-6, tumor necrosis factor (TNF), FBJ osteosarcoma oncogene (FOS) and chemokine (C-X-C motif) ligand (CXCL) 10. The top five nodes in the PPI network at 18 h (Fig. 3B) were IL-6, CCL2, heme oxygenase 1 (HMOX1), TIMP metallopeptidase inhibitor 1 (TIMP1) and serpin peptidase inhibitor clade E member 1 (SERPINE1).

### Module analysis in the network

**Figure 5:**
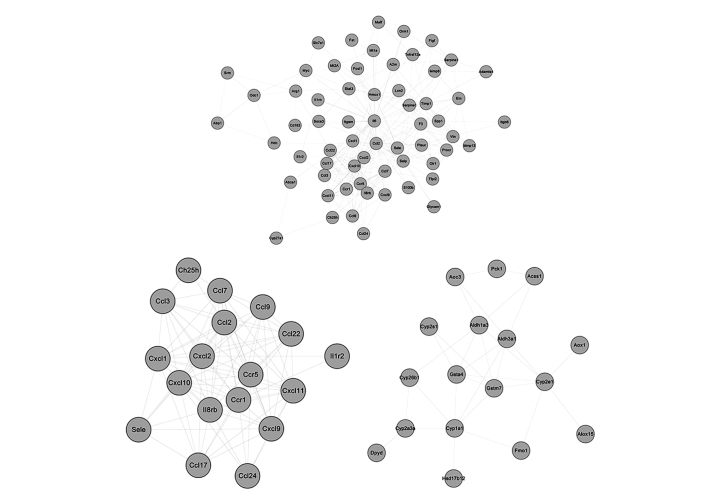
Top three modules in the protein-protein interaction network of the differentially expressed (DEGs) in pulmonary embolism at 18 h. The nodes indicate the DEGs and the edges indicate the interactions between two genes.

Since there were more nodes and edges in the PPI network at 18 h, as compared with at 2 h, ClusterONE was used to perform a module analysis for the network at 18 h only. The top three modules, which had the lowest P-values, are listed in Fig. 5. In module 1, the highest degree node was IL-6 (degree 40), and the second highest was CCL2 (degree 30). In module 2, there were seven nodes with the highest degree of 13, including CCL7, CXCL10, CXCL11, IL-8RB, CXCL2, CCL2, and chemokine (C-C motif) receptor 1 (CCR1).

### Functional enrichment analysis of modules

Following a functional enrichment analysis of the DEGs in the top three modules, the top 10 GO terms were listed (Table I). The DEGs of module 1 were significantly enriched in response to wounding (P=1.52E-14), leukocyte migration (P=5.06E-14) and the inflammatory response (P=7.05E-13); the DEGs in module 2 were markedly enriched in chemokine activity (P=8.82E-26), chemokine receptor binding (P=1.32E-25) and the immune response (P=3.53E-15); and the DEGs in module 3 were enriched in oxidation reduction (P=2.45E-10), xenobiotic metabolic process (P=1.61E-08) and iron ion binding (P=1.23E-06).

## Discussion

Due to the high mortality rate associated with PE, understanding the mechanisms of PE pathogenesis is required, in order to identify potential therapeutic targets. The present study used a gene expression profile, downloaded from GEO, to analyze the possible functions and pathways of DEGs in PE. The most significantly upregulated and downregulated genes at 2 h after PE initiation were CCL2 and Retnla, and at 18 h were TFPI2 and Cyp1a1, respectively. CCL2 was previously shown to be downregulated in a mouse model of DVT, which may be treated with IL-6 antibodies (27). Furthermore, the mRNA expression levels of chemokines, such as CCL2 and CCL7, have been shown to be significantly lower in patients with PE, as compared with controls (14). However, these findings were not in concordance with the results of the present study. CCL2 was upregulated at 2 h after microsphere injection, suggesting that the expression of CCL2 may be associated with the duration of PE; however, this requires further investigation. Previous studies have suggested that TFPI may contribute to the efficacy of low molecular weight heparins (28), which are an effective treatment for PE (29). The present study observed an upregulation of TFPI2 in PE, indicating that TFPI2 may be a target gene for the treatment of PE. The results of the present study identified novel perspectives that CCL2 may exhibit a role in the pathogenesis of PE, as well as Retnla, TFPI2 and Cyp1a1.

The chemokine signaling and cytokine-cytokine receptor interaction pathways were enriched 2 and 18 h after PE initiation, as determined by KEGG pathway analysis of the DEGs. Furthermore, the toll-like receptor signaling, nucleotide-binding oligomerization domain (NOD)-like receptor signaling and MAPK signaling pathways were all significantly enriched in the 2 h group. Cytokine-cytokine receptor interactions have previously been reported to be crucial during immunological and inflammatory responses to disease (30,31). In addition, the toll-like receptor signaling pathway may activate immune response-related pathways (32), and has been shown to be associated with chronic obstructive pulmonary disease (33). Therefore, it seems rational to speculate that the toll-like receptor signaling pathway may have a role in PE. Furthermore, the NOD-like receptor signaling pathway has been demonstrated to be associated with coagulation and inflammation (34). Further studies are required to determine whether the NOD-like receptor signaling pathway has a role in the early onset of PE.

The following DEGs: CCL2, IL-6, TNF, FOS, CXCL10, HMOX1, TIMP1 and SERPINE1, were the highest degree nodes in the PPI network. CCL7, CXCL11, Il8rb, CXCL2 and CCR1 were also observed to be significantly altered in the module analysis. The expression levels of certain TNF superfamily members have previously been shown to be significantly upregulated in patients with PE (14). Chemokines can be categorized into four classes: CXC, CC, C and CX3C, according to their structure. Lv *et al* (14), demonstrated that the mRNA expression levels of the following chemokines: CCL2, CCL7, CXCL2, CXCL10 and CXCL11, were significantly upregulated in patients with PE (14), this finding is concordant with the results of the present study. These results imply that chemokines and the TNF family may have important roles in PE. In addition, the inflammatory, defense and immune responses were significantly enriched in the DEGs, as determined by GO terms analysis. It has previously been reported that patients with PE possess a lowered immune function (35,36), and CD137L, a member of the TNF family, which is important in immune regulation (37), was significantly altered in the present study. Inflammation may also have a role in venous thromboembolism, which is comprised of DVT and PE, as it has been reported that patients with DVT present the four cardinal signs of inflammation (38,39). In the present study, the inflammatory response pathway was enriched, further indicating that inflammation may be closely associated with PE.

In conclusion, the present study analyzed the DEGs profiles of PE using a computational bioinformatics approach. A number of key genes, including CCL2 and CXCL10, TNF, Retnla, TFPI2 and Cyp1a1, were identified as having potentially crucial roles in PE. Chemokine signaling, chemokine activity pathway and inflammatory response may also be associated with the development of PE. The present study provides a novel perspective regarding the mechanisms of PE. However, further verification experiments and mechanistic studies on the process of PE are required.

## Figures and Tables

**Table I:** Biological function analysis of differentially expressed genes in the top three modules of the protein-protein interaction network.

Module	ID	Term	Count	P-value
1	GO:0009611	Response to wounding	17	1.52E-14
	GO:0050900	Leukocyte migration	10	5.06E-14
	GO:0006954	Inflammatory response	13	7.05E-13
	GO:0008009	Chemokine activity	8	3.10E-12
	GO:0042379	Chemokine receptor binding	8	3.92E-12
	GO:0005615	Extracellular space	16	1.45E-11
	GO:0006952	Defense response	14	5.25E-11
	GO:0006935	Chemotaxis	9	9.04E-11
	GO:0042330	Taxis	9	9.04E-11
	GO:0044421	Extracellular region part	17	1.57E-10
2	GO:0008009	Chemokine activity	12	8.82E-26
	GO:0042379	Chemokine receptor binding	12	1.32E-25
	GO:0005125	Cytokine activity	12	2.80E-19
	GO:0006955	Immune response	13	3.53E-15
	GO:0042330	Taxis	9	4.29E-14
	GO:0006935	Chemotaxis	9	4.29E-14
	GO:0050900	Leukocyte migration	8	3.91E-13
	GO:0005615	Extracellular space	12	1.55E-11
	GO:0007626	Locomotory behavior	9	7.66E-11
	GO:0006954	Inflammatory response	9	1.08E-10
3	GO:0055114	Oxidation reduction	11	2.45E-10
	GO:0006805	Xenobiotic metabolic process	5	1.61E-08
	GO:0009410	Response to xenobiotic stimulus	5	2.65E-08
	GO:0005506	Iron ion binding	7	1.23E-06
	GO:0009055	Electron carrier activity	6	7.78E-06
	GO:0005783	Endoplasmic reticulum	8	2.09E-05
	GO:0048037	Cofactor binding	6	3.39E-05
	GO:0005789	Endoplasmic reticulum membrane	5	1.18E-04
	GO:0042175	Nuclear envelope-endoplasmic reticulum network	5	1.49E-04
	GO:0042573	Retinoic acid metabolic process	3	1.71E-04

GO, gene ontology.
